# The effect of nationwide quarantine on anxiety levels during the COVID‐19 outbreak in China

**DOI:** 10.1002/brb3.1938

**Published:** 2020-11-11

**Authors:** Jing Zhu, Li Su, Yi Zhou, Juan Qiao, Wei Hu

**Affiliations:** ^1^ Department of Psychiatry The Affiliated Xuzhou Eastern Hospital of Xuzhou Medical University Xuzhou China; ^2^ Department of Psychiatry Xuzhou Medical University Xuzhou China; ^3^ CAS Key Laboratory of Behavior Science Institute of Psychology Chinese Academy of Sciences Beijing China; ^4^ Department of Psychology University of Chinese Academy of Sciences Beijing China; ^5^ Department of Neurobiology Army Medical University Chongqing China; ^6^ Chongqing Key Laboratory of Neuroscience Chongqing China

**Keywords:** anxiety, COVID‐19, quarantine, quarantine duration, survey

## Abstract

**Background:**

In the recent outbreak of COVID‐19, many countries have enacted various kinds of quarantine measures to slow down the explosive spread of COVID‐19. Although these measures were proven to be successful in stopping the outbreak in China, the potential adverse effects of countrywide quarantine have not been thoroughly investigated.

**Methods:**

In this study, we performed an online survey to evaluate the psychological effects of quarantine using the Zung Self‐rating Anxiety Scale in February 2020 when the outbreak had nearly peaked in China. Along with the anxiety scores, limited personal information, such as age, gender, region, education, occupation, and specifically, the type and duration of quarantine, was collected for analysis.

**Results:**

From a total of 992 valid questionnaires from 23 provinces in China, clinically significant anxiety symptoms were observed in 9.58% of respondents according to clinical diagnostic standards in China. The specific groups of people showing higher levels of anxiety were (a) adolescents (<18 years); (b) respondents with education lower than junior high school; (c) people with chronic diseases; and (d) frontline medical personnel. Other characteristics, such as gender, marital status, region, and acquaintance with suspected or confirmed cases of COVID‐19, did not affect anxiety levels significantly. Respondents who experienced different forms of quarantine showed different anxiety levels. People undergoing centralized quarantine have higher levels of anxiety. Unexpectedly, longer durations of quarantine did not lead to a significant increase in anxiety level.

**Conclusions:**

Our results suggest a rather mild psychological influence caused by the countrywide quarantine during the COVID‐19 outbreak in China and provide a reference for other countries and regions battling COVID‐19.

## Graphical abstract

52.42% of the respondents experienced quarantine duration more than 2 weeks, and 9.58% of the respondents showed clinical significance of anxiety (SAS > 50). Medical/non‐medical frontline staffs need more psychological care.Prolonged duration of quarantine do not largely change anxiety level.

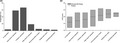

## 1 INTRODUCTION

On March 11, 2020, the World Health Organization declared SARS‐COV‐2‐induced COVID‐19 a "pandemic" during a news conference in Geneva (European Centre for Disease Prevention & Control, 2020). With more than 120,000 confirmed infections and more than 5,000 lives taken in more than 100 countries, there is still no specific medicine to cure the highly transmissible COVID‐19 (Zhang & Liu, 2020). Although some promising drugs (e.g., remdesivir, favipiravir) are currently in clinical trials (Dong et al., 2020; Wang et al., 2020), the most effective way to stop COVID‐19 by far is still the oldest way that humans have used to battle epidemics for hundreds of years: quarantine.

China has been conducting quarantine measures in many provinces throughout the country since late January, and the results are significant. In approximately two weeks, the daily number of new patients peaked and then began to decline (World Health Organization, 2020). Six weeks after quarantine, the daily number of new patients dropped to less than 100 in China (National Health Commission of the People's Republic of China, 2020a). Similar quarantine methods have also been adopted by other countries, such as the Republic of Korea. Since the beginning of regional quarantine in Daegu, the number of newly diagnosed cases in the Republic of Korea has been declining steadily as well (Choi & Ki, 2020). Other countries, such as Iran (countrywide quarantine since March 2020) and Italy (countrywide quarantine since March 2020), have also undertaken quarantine measures recently, and more countries might also take these approaches into consideration to stop the COVID‐19 outbreak.

However, the decision to quarantine was never an easy option due to predictable enormous negative impacts on the economy and unpredictable psychological harm to the quarantined population. Previous reports have shown that negative emotions caused by quarantine can lead to various kinds of consequences, such as anxiety, depression, and posttraumatic stress disorder (PTSD; Brooks et al., 2020). During the outbreak of SARS (severe acute respiratory syndrome) in 2003, an increased prevalence of depression and PTSD was found in quarantined persons (Hawryluck et al., 2004). During the MERS (Middle East respiratory syndrome) outbreak, quarantined people showed more negative emotions, such as anxiety and anger (Jeong et al., 2016). This evidence suggests that the potential psychological and other effects of quarantine could be significant and should be carefully taken into consideration.

However, unlike the quarantine measures during SARS or MERS outbreaks, a much larger population in China has been affected by the countrywide quarantine to battle COVID‐19. A rough estimation is that more than 100 million people in China were affected by this countrywide quarantine (Tian et al., 2020), a number never seen before in human history. This raises new concerns regarding the potential adverse effects of large‐scale quarantine. Will countrywide quarantine cause broad social panic and even chaos? Conversely, super large‐scale quarantine might have no major impacts on the general public because everyone was quarantined, according to the theory of social comparison processes (Festinger, 1954). These controversial questions cannot be easily answered without systematic investigation but could be critical for future decisions regarding large‐scale quarantine.

To quickly understand the psychological impact that the COVID‐19 quarantine measures may bring to people, researchers have performed many investigations. However, the results are not consistent. As far as China is concerned, some studies have shown that the incidence of depression and anxiety among quarantined respondents is significantly higher than that of nonquarantined respondents (Tang et al., 2020; Zhao et al., 2020). However, another study from China suggests that psychological problems during the epidemic have nothing to do with quarantine control measures but rather are related to the impact of the epidemic on daily life (Zhu, Wu, et al., 2020). The survey results for the population of different countries are also inconsistent. Researchers in Israel found that respondents who underwent quarantine measures showed only low levels of anxiety (Horesh et al., 2020). Some studies indicate that quarantine leads to increased tension in young people (Ozamiz‐Etxebarria et al., 2020; Tee et al., 2020). In contrast, Irish researchers found that older people in quarantine have increased anxiety (Hyland et al., 2020). Therefore, further investigation of the psychological impact of quarantine measures is necessary.

In this study, we performed an online questionnaire survey during the middle stage of the COVID‐19 outbreak in China (Figure S1) to understand the psychological effect on quarantined persons using the Zung Self‐rating Anxiety Scale (Zung, 1971). Along with the anxiety scores, limited personal information, such as age, gender, region, education, etc. and specifically, the type and duration of quarantine, was collected for analysis. We further assessed the influence of different types and durations of quarantine used in China. Our results provide new insights into the experiences of quarantined persons during the COVID‐19 outbreak in China, which could be important for nationwide containment of COVID‐19 in other countries.

## 2 METHOD

### 2.1 Survey tool

The survey was conducted in the form of an online questionnaire between 12:00 p.m. February 19, 2020, and 12:00 p.m. February 26, 2020 (UTC + 8), and consisted of a general information survey and a Zung Self‐rating Anxiety Scale (SAS; Zung, 1971). The general survey included (a) basic information of the respondent, such as gender, age, region, education level, occupation, marital status and phone number (optional) for further contact and (b) quarantine‐related information on the respondent during the COVID‐19 outbreak. Anxiety assessment was performed using the Chinese version of the SAS compiled by William W. K. Zung. The SAS is used to measure the subjective anxiety of subjects using a 4‐point scale: no or very little time, a small amount of time, a considerable amount of time, and most or all of the time. Raw scores were then converted to index scores following a previous report (Zung, 1971). An index score equal to or larger than 50 is considered to indicate the clinical significance of anxiety (Zung, 1986): 50–59 is mild anxiety, 60–69 is moderate anxiety, and 70 or more is severe anxiety. The Zung SAS was first introduced in China and translated into Chinese in 1984 by Dr. Zhengyu Wang from the Shanghai Institute of Mental Health (Wang & Chi, 1984). In 1986, Professor Zisi Dai, an expert from the China National Rating Scale Association, took the lead in establishing the Chinese norm of SAS. After that, Chinese clinicians and researchers extensively used the Chinese version of the Zung SAS (Liu et al., 1995; Wang & Xu, 2009). It has also been included in the "Manual of Psychiatric Rating Scales" (2015, Second Edition, edited by Dr. Mingyuan Zhang and Dr. Yanling He, Hunan Science and Technology Press). In our hospital alone, the Chinese version of the Zung SAS has been used more than 200,000 times for the clinical diagnosis of anxiety in the last three years.

### 2.2 Investigation methods

The online survey was performed using the professional online survey service Questionnaire Star (https://www.wjx.cn) and then released nationwide through social media software (such as WeChat, Weibo, QQ, etc.). This study was conducted with the respondent's informed consent (including assent/parental consent for adolescents) and approved by the ethics committee of The Affiliated Xuzhou Eastern Hospital of Xuzhou Medical University.

### 2.3 Quality control

IP addresses are often used for quality control in online surveys. Considering that quarantined personnel or families might share the same internet and same IP address, we did not limit the number of questionnaires from the same IP address. Instead, we performed a post hoc check to ensure the reliability of the questionnaires. The target was to recruit 811 participants based on a sample size calculation with a 90% confidence level, 5% significance level, and 5% margin of error around the previously reported prevalence of anxiety disorder in China (Huang et al., 2019). For a total of 997 questionnaires collected, 5 were identified as invalid questionnaires due to abnormal key data (e.g., 4 years old). For a total of 888 unique IP addresses, 816 (82.26%) IP addresses filed 1 questionnaire, 55 (6.19%) filed 2, 10 (1.13%) filed 3, and 7 (0.79%) filed 4 or more (max 7). The median time to finish this questionnaire was 291 s, with an interquartile range from 215–415 s.

### 2.4 Statistical methods

Statistical analysis was performed using SPSS 22 (IBM). Cronbach's alpha was used to measure the reliability. The Kaiser–Meyer–Olkin (KMO) value and Bartlett's sphericity test were used to examine the suitability of the data. Independent sample *t* test was used for analysis of gender, region, health status, and acquaintance with suspected or confirmed cases of COVID‐19. For multiple groups of samples (age, education, marital status, and personnel category), one‐way ANOVA was used. The confidence level was set at *p* < .05 unless specified.

## 3 RESULT

### 3.1 Demographics and description of respondents

For the questionnaire survey, 992 valid questionnaires (see Methods for details) were collected between 12:00 p.m. February 19, 2020 and 12:00 p.m. February 26, 2020 (UTC + 8). Cronbach's alpha was equal to 0.812, suggesting that the reliability was robust. Moreover, the KMO value was 0.917, indicating that the sampling was adequate. Bartlett's sphericity test was considered statistically significant with a *p* value smaller than .001.

Table 1 summarizes personal characteristics from the valid questionnaires. There were 424 males and 568 females. The age ranged from 11 to 75 years, with a median age of 36 years (IQR: interquartile range, 28–42). The respondents were from 23 provinces; 214 were (21.6%) from Hubei Province, the province with the most severe outbreak (>60,000 infections), and 778 (78.4%) were from other regions in China. The education levels of 75 (7.6%) respondents were junior high school or below; 177 (17.8%), high school/technical secondary school; 247 (24.9%), college/higher vocational; 423 (42.6%), undergraduate; and 70 (7.1%), postgraduate. In terms of marital status, 700 were married (70.6%), 252 were unmarried (25.4%), 37 were divorced (3.7%), and 3 were widowed (0.3%). For the personnel category, 41 (4.1%) and 102 (10.3%) were frontline medical and nonmedical personnel for battling COVID‐19, respectively, 106 (10.7%) were non‐frontline medical personnel, and 743 (74.9%) were other personnel. In terms of health status, 961 (96.9%) were healthy, and 31 (3.1%) had chronic diseases. Among all respondents, 81 (8.2%) had acquaintance with people diagnosed with or suspected of having COVID‐19, and 911 (91.8%) were not.

**TABLE 1:** Characteristics of quarantined persons who responded to the survey

Characteristic	No. (%) (*N* = 992)
Gender	
Male	424 (42.7)
Female	568 (57.3)
Age (y)	
<18	12 (1.2)
18–34	449 (45.3)
35–49	411 (41.4)
50–64	91 (9.2)
≥65	29 (2.9)
Region	
Within Hubei	214 (21.6)
Outside Hubei	778 (78.4)
Education	
Junior high school and below	75 (7.6)
High school/secondary school	177 (17.8)
College/higher vocational	247 (24.9)
Undergraduate	423 (42.6)
Postgraduate	70 (7.1)
Marital status	
Married	700 (70.6)
Unmarried	252 (25.4)
Divorced	37 (3.7)
Widowed	3 (0.3)
Personnel category	
New crown pneumonia frontline medical staff	41 (4.1)
Other frontline staff	102 (10.3)
Other medical staff	106 (10.7)
Other people besides the above	743 (74.9)
Health status	
Health	961 (96.9)
Have other diseases besides COVID‐19	31 (3.1)
Acquaintance with suspected or confirmed cases of COVID‐19	
Yes	81 (8.2)
No	911 (91.8)

### 3.2 Prevalence of anxiety symptoms by demographics of respondents

Table 2 summarizes the prevalence of anxiety score according to demographics of respondents. According to the clinical diagnosis standards in China and previous reports (Zung, 1986), 897 (90.42%) had normal SAS scores (<50), and 95 (9.58%) had elevated scores (≥50), indicating the clinical significance of anxiety. Moreover, of these, 67 (6.75%) had mild anxiety, 20 (2.02%) had moderate anxiety, and 8 (0.81%) had severe anxiety. Anxiety scores in different age (*F* = 3.168, *p* = .013), education (*F* = 3.865, *p* = .004), health status (*t* = −3.043, *p* = .005), and personnel category (*F* = 5.802, *p* = .001) groups were statistically significant (*p* < .05). Adolescents (mean = 46.33, median = 45.50, IQR 32–62), respondents with education lower than junior high school (mean = 41.11, median = 41, IQR 35–45), people with chronic diseases (mean = 44.06, median = 42, IQR 36–51), and frontline medical personnel (mean = 42.61, median = 41, IQR 34–44) had higher anxiety scores than other groups, which is consistent with an earlier report (Zhu, Xu, et al., 2020).

**TABLE 2:** Prevalence of anxiety symptoms by patient demographics

Characteristic	No. (%) (*N* = 992)
Prevalence	
SAS	
<50	897 (90.42)
≥50	95 (9.58)

Classification	Mean	*SD*	*p* value
Gender			
Male	38.96	9.440	.132
Female	38.09	8.345	
Age (y)			
<18	46.33	16.228	.013
18–34	38.53	8.261	
35–49	38.03	9.081	
50–64	38.30	8.337	
≥65	40.79	10.094	
Region			
Within Hubei	39.14	8.692	.205
Outside Hubei	38.28	8.871	
Education			
Junior high school and below	41.11	10.120	.004
High school/secondary school	38.94	7.977	
College/higher vocational	39.08	8.602	
Undergraduate	37.35	8.207	
Postgraduate	38.93	12.489	
Marital status			
Married	38.24	8.492	.678
Unmarried	38.97	9.734	
Divorced	39.19	9.027	
Widowed	38.67	7.767	
Personnel category			
COVID‐19 frontline medical staff	42.61	13.773	.001
Other frontline staff	39.95	9.010	
Other medical staff	36.55	7.967	
Other people besides the above	38.30	8.497	
Health status			
Health	38.28	8.725	.005
Have other diseases besides COVID‐19	44.06	10.466	
Acquaintance with suspected or confirmed cases of COVID‐19			
Yes	39.90	10.359	.126
No	38.33	8.682	

Anxiety scores between different genders (*t* = 1.508, *p* = .132), regions (*t* = 1.269, *p* = .205), marital status (*F* = 0.506, *p* = .678), and people acquainted with suspected or confirmed COVID‐19 patients (*t* = −1.531, *p* = .126) were not significantly different among the respondents. The mild and not significant difference between respondents inside and outside Hubei Province was not expected because Hubei Province has more than 80% of the infections in the whole country (National Health Commission of the People's Republic of China, 2020b).

### 3.3 Different types of quarantine affect anxiety level differently

**FIGURE 1:**
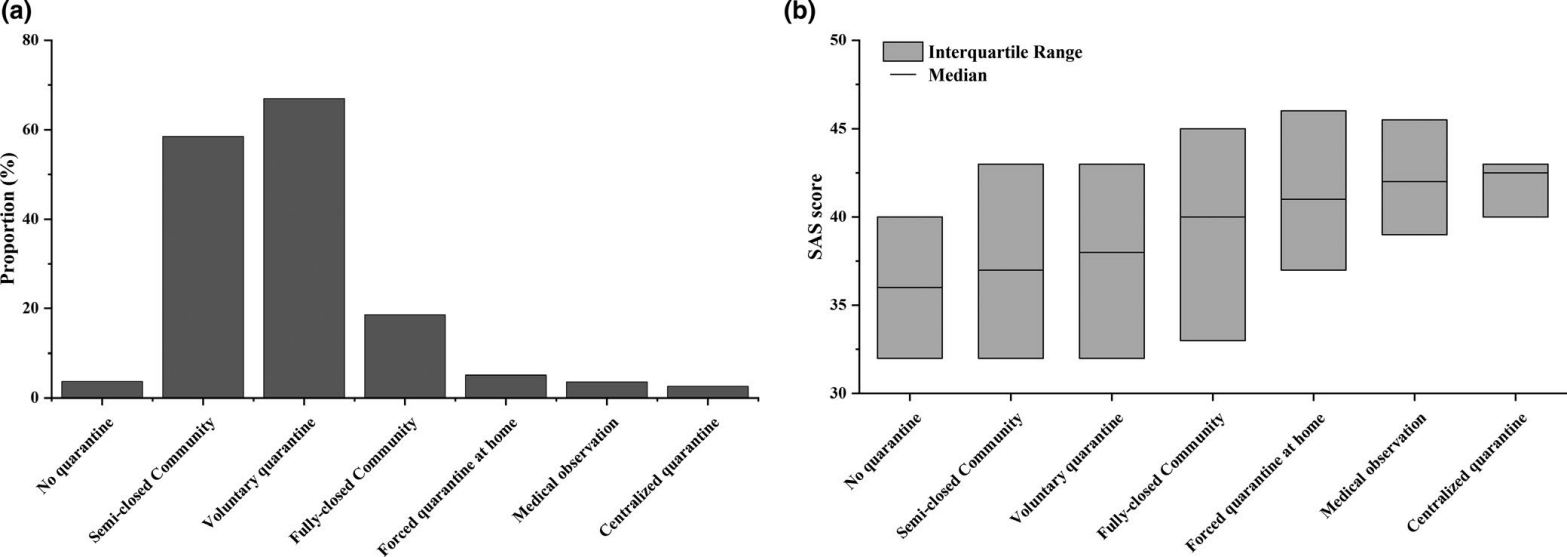
Proportion and anxiety scores of six quarantine groups. (a) Proportion of six types of quarantine groups. (b) Box plot of anxiety scores in each quarantine group. The interquartile range represents the range of 25%–75% scores

Multiple types of quarantine have been used during the COVID‐19 outbreak in China. Figure 1 shows the proportion and SAS scores of respondents who experienced different kinds of quarantines. Six kinds of quarantines were investigated in this survey: (a) Voluntary quarantine: stay at home voluntarily; (b) Semiclosed community: a permit is delivered to residents for a limited number of entries and exits; (c) Fully closed community: no one is allowed to enter or leave except for personnel responsible for supply dispensing; (d) Forced quarantine: required to stay at home for certain periods; (e) Centralized quarantine: quarantine at a designated place (e.g., a hotel); (f) Medical observation: quarantine at a designated hospital. Because one person could experience multiple types of quarantines, his/her data might appear in different groups. Most respondents (955/992, 96.27%) had at least one type of quarantine experience (Figure 1a), and only 37/992 (3.73%) had no quarantine experience at all. Further analysis revealed that the majority (29/37, 78.38%) of respondents without quarantine experience are frontline personnel for battling COVID‐19, either medical or nonmedical.

Statistical results showed a significant difference (Figure 1b, *F* = 5.132, *p* < .001) between different types of quarantine: no quarantine (median = 36, IQR 31.5–40), semiclosed community (median = 37, IQR 32–43), voluntary quarantine (median = 38, IQR 32–43), fully closed community (median = 40, IQR 33–45), forced quarantine at home (median = 41, IQR 37–46), medical observation (median = 42, IQR 38.5–45.75), and centralized quarantine (median = 42.5, IQR 39.25–43.5). Among them, those who received centralized quarantine had the highest anxiety level. This suggested that after quarantine, this specific population might need medical assistance from professional psychologists.

### 3.4 Duration of quarantine does not significantly increase anxiety level

**FIGURE 2:**
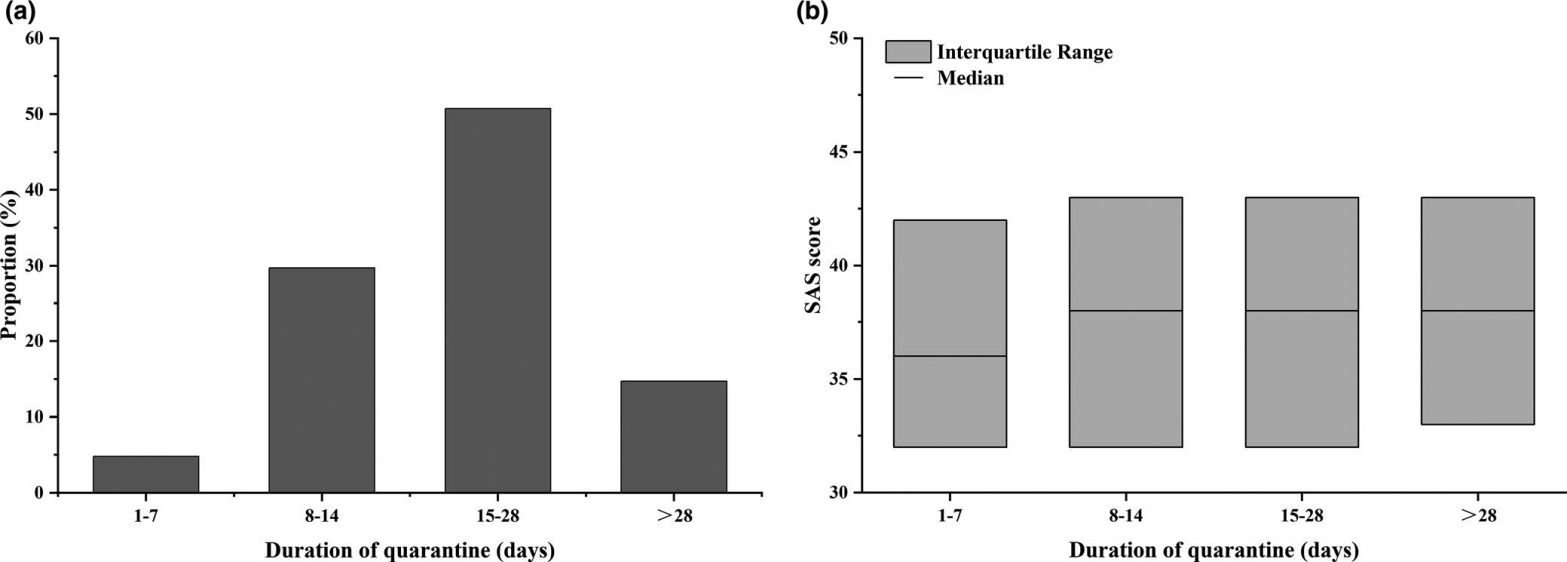
Proportion and anxiety scores of four groups of quarantine duration. (a) Proportion of different durations of quarantine. (b) Box plot of anxiety scores in each group of quarantine duration. The interquartile range represents the range of 25%–75% scores

Previous studies suggested that a longer duration of quarantine can result in worse psychological impacts (Brooks et al., 2020). Figure 2 shows the proportion and SAS scores of people with different quarantine durations. Because the survey was conducted in middle‐late February and countrywide quarantine started in late January, most respondents (65.49%) had been quarantined for more than two weeks. Only 4.79% of respondents had a quarantine duration of less than 7 days. Statistical results revealed a mild but not significant difference between anxiety scores in groups with different durations (Figure 2b, *F* = 1.644, *p* = .178). The lowest score was found in the group with less than 7 days (mean = 36.87, median = 36, IQR 32–42). However, for durations longer than 7 days, all three groups had very similar scores: 8–14 days (mean = 38.14, median = 38, IQR 32–43), 15–28 days (mean = 39.04, median = 38, IQR 32–43), and longer than 28 days (mean = 39.83, median = 38, IQR 33–43). This unexpected finding is encouraging but could be the result of many different reasons, which we will discuss in detail later.

## 4 DISCUSSION

Through this study, we found that countrywide quarantine in China does lead to an increase in anxiety levels in certain populations. For example, frontline personnel for battling COVID‐19 (medical or nonmedical) have an increased risk of anxiety. These results are consistent with previous literature (Lee et al., 2018; Tam et al., 2004). The results of some concurrent studies have also confirmed this finding (Giallonardo et al., 2020; Maciaszek et al., 2020). The results of this study also show that the following specific groups of people showed increased levels of anxiety in their respective groups: (a) adolescents; (b) respondents with education lower than junior high school; and (c) people with chronic diseases. This is also consistent with the survey results in the same period (Guo et al., 2020; Khan et al., 2020; Lei et al., 2020; Rossi et al., 2020; Wong et al., 2020). Therefore, we suggest that further psychological assistance should be provided to specific populations.

However, some research results give different conclusions. One study showed that mental health problems have nothing to do with quarantine control measures, but instead the impact on daily life (Zhu, Wu, et al., 2020). This may be related to different investigation times and evaluation standards. The investigation time of their study was from February 12 to March 17, 2020. The time was relatively long, covering the peak and recovery periods of COVID‐19 in China, so the interviewees' psychological changes were also relatively large. Moreover, the impact on daily life itself is a subjective impression and lacks objective evaluation criteria.

Another study showed that the elderly individuals (≥65 years) in the quarantine area have increased levels of COVID‐19‐related anxiety (Hyland et al., 2020). We believe that this may be related to the survey's time, age structure, evaluation tool, and respondents' culture. Their investigation began in the first week of Ireland's national quarantine (March 31‐April 5, 2020). Although COVID‐19 had been raging around the world for several months, when the epidemic was indeed sensed, everyone needed to adapt, especially the elderly population, because this age group is particularly vulnerable to COVID‐19‐related mortality (Zhou et al., 2020). Moreover, the proportion of older adults participating in this study was relatively high (12.20%), reflecting more of their mental state. The study also used the Generalized Anxiety Disorder Scale‐7 (GAD‐7) to assess the respondents' anxiety state, which may differ from the SAS assessment results. Additionally, cultural differences may affect the results of the survey.

Our research also investigated the impact of different quarantine types on the population's psychology for the first time. It turns out that compared with people who experienced other quarantine types, people who received centralized quarantine had the highest anxiety levels. This may be related to the respondents' uncertainty about their health: They have had close contact with patients with confirmed COVID‐19, but they have not shown positive symptoms. Therefore, they are apprehensive about being infected. Moreover, centralized quarantine measures have caused these investigators to leave their familiar environment and family members. During the quarantine period, the quarantined person can stay alone in only a strange and crowded room. In addition, the person cannot talk with anyone, except via the only tool to communicate with the outside world‐the phone. Loneliness contributes to anxiety (Gonzalez‐Sanguino et al., 2020).

Although our research found that the length of time under quarantine has no noticeable effect on anxiety, some studies have found that COVID‐19‐related stress has a delayed effect (Gan et al., 2020). By analyzing some recent studies, we found that this effect seems to exist, even though the survey population and tools were different (Liu et al., 2020; Shi et al., 2020). This finding is thought to be explained by the "psychological typhoon eye effect." Our research results fill knowledge at a specific point in time, thereby showing the public's psychological changes and trends under quarantine conditions.

In addition, our research results show that quarantine‐related anxiety is relatively low, which is different from the results of another Italian study. They found that quarantine measures can cause high stress and distress (Casagrande et al., 2020). However, the findings of our results could be unique due to the following conditions in which the study was conducted. (a) The survey was conducted between February 19 and February 26, a time window where the outbreak of COVID‐19 had been largely controlled in China (Figure S1). At this stage, the initial panic of the COVID‐19 outbreak has gone, and many people in the country are still under quarantine. This ensured a more accurate reflection of the psychological influence induced by quarantine, which is the main purpose of this study. However, it is still unclear what happened in the first a few days since the outbreak. (b) Nonetheless, in the early stages of quarantine measures, inadequate hand sanitizer supplies caused anxiety among certain populations such as college students (Li et al., 2020). With the rapid increase in market supply and the gradual decline in the price index, the shortage of medical resources and food supply was effectively alleviated. Financial aid was also beneficial, including fully covered costs for diagnosis and treatment. Other appraoches such as online psychotherapy (Ho et al., 2020) could also be crucially important in reducing social panic risk. (c) Very different from previous epidemic outbreaks such as SARS in 2003, the whole world has become an information society in the past 20 years. Whether voluntary or forced quarantine, people are still well connected with the outside world through the Internet and smart phones. (d) As we mentioned earlier, the effect of social comparison processes theory could also be helpful for easing nervousness and other negative emotions during quarantine. These 4 points can explain why anxiety levels were found to be low in this study. Even though the quarantine anxiety level in Israel is low, the causes are not precisely the same. Possible reasons include the relative resilience of Israeli society. Many Israelis have experienced decades of war and continued political violence, so they may be used to coping with stress (Horesh et al., 2020).

In the human history of fighting epidemics, there are very few successful cases in which the outbreak was stopped by specific drugs or vaccines. This is usually caused by the rapid outbreak and the relatively slow development of specific drugs. When many companies finally developed SARS vaccines that could be used in clinical trials, the epidemic was almost complete (Jiang et al., 2005). On the one hand, we should keep developing effective drugs and therapies for COVID‐19 as soon as possible; on the other hand, we should also consider using reasonable and effective quarantine methods (regional or national) to slow down the spread of the epidemic and buy some time for pharmaceutical research and production.

The more encouraging part of the results is that compared with previous studies of quarantine (Jeong et al., 2016; Reynolds et al., 2008), the super large‐scale quarantine in China did not lead to a significant change in anxiety level. The level of anxiety in Hubei Province, the most severely infected area in the world, was not significantly but only slightly higher than that outside Hubei Province (*p* = .205). According to Festinger's social comparison theory, individuals in a group tend to compare themselves with other people to determine their self‐worth. When the quarantined people were compared and found that they were in the same quarantined situation, their inner sense of value returned to its place, alleviating the quarantine's psychological anxiety. Considering that Hubei Province accounts for more than 80% of total infections in China and was the first quarantined province, these results are promising to people in severely infected and strictly controlled areas such as Daegu in the Republic of Korea or Lombardy in Italy.

The limitations of our study and implications are as follows. (a) Compared with the super large‐scale number of people affected by the countrywide quarantine, the sample size of our survey was very small. Thus, our results might not be able to fully reveal the true situation. (b) Although there is not much we can do, the form of online surveys naturally ignores certain populations, especially elderly adults who could be more vulnerable to COVID‐19 (Wu & McGoogan, 2020) but do not use smart phones. In our survey, only a very small portion of respondents (2.9%) were older than 65 years. (c) When designing the study, we paid attention to the impact of physical health on mental state, ignoring the possibility of the respondents' previous mental disorders, which may affect the result. (d) The COVID‐19 epidemic was a sudden surprise to everyone. We do not know who will be susceptible to quarantine measures, so we have no prequarantine scores for comparison. The current research design cannot be used to evaluate the impact of quarantine on anxiety levels.

As we mentioned earlier, anxiety levels could be a mixed result of many different factors. It is almost impossible to determine the pure contribution of quarantine to anxiety level. For example, worries about potential infection or financial problems could also elevate anxiety scores (McAlonan et al., 2007; Zheng et al., 2005). Some researchers found that anxiety was associated with a longer duration of home study in China (Wang, Pan, Wan, Tan, Xu, Ho et al., 2020; Wang, Pan, Wan, Tan, Xu, McIntyre, et al., 2020). Problematic smartphone use may also cause increased anxiety (Elhai et al., 2020). Some respondents even left messages to express their concerns about childcare and education due to school suspensions.

## CONFLICT OF INTEREST

The authors declare that they have no conflicts of interest.

## AUTHOR CONTRIBUTION

The authors' contributions are as follows: Jing Zhu and Wei Hu conceived and developed the idea for the paper and revised the manuscript; Li Su contributed to data collection and wrote numerous drafts; Juan Qiao contributed to data analysis and interpretation of the data. Yi Zhou provided advice and assistance with statistical operations and translating. All authors read and approved the final manuscript.

## ETHICAL STATEMENTS

The authors assert that all procedures contributing to this work comply with the ethical standards of the relevant national and institutional committees on human experimentation and with the Helsinki Declaration of 1975, as revised in 2008. This study was conducted with informed consent of the respondents and has been approved by the ethics committee of The Affiliated Xuzhou Eastern Hospital of Xuzhou Medical University. Questionnaires were collected anonymously to ensure that personal privacy was not disclosed. Secure encryption provided by "Questionnaire Star" was performed throughout the entire process, including data collection, transmission, and release.

### Peer Review

The peer review history for this article is available at https://publons.com/publon/10.1002/brb3.1938.

## Supporting information

Fig S1

## Data Availability

The data used in this study are available on request from the corresponding author.
